# Supplementary material for: Intragenus (Homo) variation in a chemokine receptor gene (CCR5)
Source: PLoS One. 2018 Oct 2;13(10):e0204989. doi: 10.1371/journal.pone.0204989 (PMC6168169; doi:10.1371/journal.pone.0204989)
Supplement: S1 Table — (DOCX) [file pone.0204989.s001.docx]

| **Supplementary Table1: Introgression data** | | | |  |  |  |  |
| --- | --- | --- | --- | --- | --- | --- | --- |
| **SNP** | **GenPos** | **Neandertal DAF** | **YRI DAF** | **N derived alleles** | **Av post p of N** | **DAF** | **N hap freq** |
| 3:46409636 | 67.612 | 0 | 0.19 | 1 | -1 | 0.00 | 0 |
| 3:46409728 | 67.612 | 0 | 0.00 | 1 | -1 | 0.00 | 0 |
| 3:46409935 | 67.612 | 0 | 0.00 | 1 | -1 | 0.00 | 0 |
| 3:46410020 | 67.612 | 0 | 0.02 | 0 | -1 | 0.00 | 0 |
| 3:46410036 | 67.612 | 0 | 0.04 | 335 | -1 | 0.25 | 0 |
| 3:46410137 | 67.612 | 0 | 0.00 | 101 | -1 | 0.08 | 0 |
| 3:46410279 | 67.612 | 0 | 0.00 | 4 | -1 | 0.00 | 0 |
| 3:46410306 | 67.612 | 0 | 0.37 | 0 | -1 | 0.00 | 0 |
| 3:46410396 | 67.612 | 0 | 0.00 | 0 | -1 | 0.00 | 0 |
| 3:46410494 | 67.612 | 0 | 0.06 | 594 | -1 | 0.45 | 0 |
| 3:46410746 | 67.612 | 0 | 0.00 | 2 | -1 | 0.00 | 0 |
| 3:46410768 | 67.612 | 0 | 0.00 | 1 | -1 | 0.00 | 0 |
| 3:46410921 | 67.612 | -1 | 0.00 | 1 | -1 | 0.00 | 0 |
| 3:46410932 | 67.612 | 0 | 0.00 | 1 | -1 | 0.00 | 0 |
| 3:46410936 | 67.612 | 0 | 0.06 | 573 | -1 | 0.43 | 0 |
| 3:46410938 | 67.612 | 0 | 0.02 | 0 | -1 | 0.00 | 0 |
| 3:46410953 | 67.612 | 0 | 0.02 | 0 | -1 | 0.00 | 0 |
| 3:46410962 | 67.612 | 0 | 0.02 | 0 | -1 | 0.00 | 0 |
| 3:46411115 | 67.612 | 0 | 0.00 | 0 | -1 | 0.00 | 0 |
| 3:46411133 | 67.612 | 0 | 0.37 | 0 | -1 | 0.00 | 0 |
| 3:46411174 | 67.612 | 0 | 0.01 | 0 | -1 | 0.00 | 0 |
| 3:46411200 | 67.612 | -1 | 0.01 | 0 | -1 | 0.00 | 0 |
| 3:46411530 | 67.612 | 0 | 0.00 | 1 | -1 | 0.00 | 0 |
| 3:46411542 | 67.612 | 0 | 0.11 | 452 | -1 | 0.34 | 0 |
| 3:46411661 | 67.612 | 0 | 0.04 | 105 | -1 | 0.08 | 0 |
| 3:46411755 | 67.612 | -1 | 0.01 | 0 | -1 | 0.00 | 0 |
| 3:46411817 | 67.612 | 0 | 0.00 | 0 | -1 | 0.00 | 0 |
| 3:46411840 | 67.612 | 0 | 0.43 | 577 | -1 | 0.43 | 0 |
| 3:46411846 | 67.612 | 0 | 0.00 | 2 | -1 | 0.00 | 0 |
| 3:46411935 | 67.612 | 0 | 0.30 | 657 | -1 | 0.49 | 0 |
| 3:46412138 | 67.613 | 0 | 0.00 | 0 | -1 | 0.00 | 0 |
| 3:46412149 | 67.613 | 0 | 0.00 | 2 | -1 | 0.00 | 0 |
| 3:46412244 | 67.613 | 0 | 0.01 | 0 | -1 | 0.00 | 0 |
| 3:46412259 | 67.613 | 0 | 0.31 | 657 | -1 | 0.49 | 0 |
| 3:46412262 | 67.613 | 0 | 0.35 | 0 | -1 | 0.00 | 0 |
| 3:46412308 | 67.613 | 0 | 0.06 | 579 | -1 | 0.44 | 0 |
| 3:46412554 | 67.613 | 0 | 0.01 | 0 | -1 | 0.00 | 0 |
| 3:46412559 | 67.613 | 0 | 0.18 | 205 | -1 | 0.15 | 0 |
| 3:46412585 | 67.613 | 0 | 0.00 | 0 | -1 | 0.00 | 0 |
| 3:46412708 | 67.613 | 0 | 0.26 | 1 | -1 | 0.00 | 0 |
| 3:46412753 | 67.613 | 0 | 0.00 | 3 | -1 | 0.00 | 0 |
| 3:46412930 | 67.613 | 0 | 0.26 | 1 | -1 | 0.00 | 0 |
| 3:46412978 | 67.613 | 0 | 0.02 | 0 | -1 | 0.00 | 0 |
| 3:46413064 | 67.613 | -1 | 0.00 | 0 | -1 | 0.00 | 0 |
| 3:46413418 | 67.613 | 0 | 0.06 | 577 | -1 | 0.43 | 0 |
| 3:46413419 | 67.613 | 0 | 0.06 | 0 | -1 | 0.00 | 0 |
| 3:46413439 | 67.613 | 0 | 0.00 | 3 | -1 | 0.00 | 0 |
| 3:46413623 | 67.613 | 0 | 0.00 | 2 | -1 | 0.00 | 0 |
| 3:46413632 | 67.613 | 0 | 0.00 | 0 | -1 | 0.00 | 0 |
| 3:46413655 | 67.613 | 0 | 0.01 | 0 | -1 | 0.00 | 0 |
| 3:46413676 | 67.613 | -1 | 0.01 | 19 | -1 | 0.01 | 0 |
| 3:46413743 | 67.613 | 0 | 0.06 | 577 | -1 | 0.43 | 0 |
| 3:46413781 | 67.613 | -1 | 0.00 | 1 | -1 | 0.00 | 0 |
| 3:46413798 | 67.613 | -1 | 0.00 | 3 | -1 | 0.00 | 0 |
| 3:46413889 | 67.613 | 0 | 0.00 | 2 | -1 | 0.00 | 0 |
| 3:46413932 | 67.613 | 0 | 0.02 | 0 | -1 | 0.00 | 0 |
| 3:46413943 | 67.613 | -1 | 0.00 | 6 | -1 | 0.00 | 0 |
| 3:46413950 | 67.613 | -1 | 0.06 | 577 | -1 | 0.43 | 0 |
| 3:46414199 | 67.613 | 0 | 0.00 | 0 | -1 | 0.00 | 0 |
| 3:46414235 | 67.613 | 0 | 0.00 | 0 | -1 | 0.00 | 0 |
| 3:46414281 | 67.613 | 1 | 0.74 | 1329 | -1 | 1.00 | 0 |
| 3:46414282 | 67.613 | 0 | 0.00 | 0 | -1 | 0.00 | 0 |
| 3:46414339 | 67.613 | 0 | 0.00 | 0 | -1 | 0.00 | 0 |
| 3:46414349 | 67.613 | 0 | 0.00 | 3 | -1 | 0.00 | 0 |
| 3:46414384 | 67.613 | 0 | 0.00 | 2 | -1 | 0.00 | 0 |
| 3:46414436 | 67.613 | 0 | 0.00 | 1 | -1 | 0.00 | 0 |
| 3:46414470 | 67.613 | 0 | 0.00 | 2 | -1 | 0.00 | 0 |
| 3:46414485 | 67.613 | 0 | 0.00 | 1 | -1 | 0.00 | 0 |
| 3:46414498 | 67.613 | 0 | 0.00 | 0 | -1 | 0.00 | 0 |
| 3:46414529 | 67.613 | -1 | 0.01 | 0 | -1 | 0.00 | 0 |
| 3:46414557 | 67.613 | 0 | 0.00 | 24 | -1 | 0.02 | 0 |
| 3:46414580 | 67.613 | 0 | 0.00 | 0 | -1 | 0.00 | 0 |
| 3:46414593 | 67.613 | 0 | 0.00 | 1 | -1 | 0.00 | 0 |
| 3:46414618 | 67.613 | 0 | 0.02 | 0 | -1 | 0.00 | 0 |
| 3:46414696 | 67.613 | 0 | 0.00 | 0 | -1 | 0.00 | 0 |
| 3:46414709 | 67.613 | 0 | 0.00 | 2 | -1 | 0.00 | 0 |
| 3:46414712 | 67.613 | 0 | 0.01 | 0 | -1 | 0.00 | 0 |
| 3:46414731 | 67.613 | 0 | 0.00 | 0 | -1 | 0.00 | 0 |
| 3:46414753 | 67.613 | 0 | 0.00 | 0 | -1 | 0.00 | 0 |
| 3:46414766 | 67.613 | 0 | 0.00 | 0 | -1 | 0.00 | 0 |
| 3:46414775 | 67.613 | 0 | 0.00 | 0 | -1 | 0.00 | 0 |
| 3:46414925 | 67.613 | 0 | 0.00 | 1 | -1 | 0.00 | 0 |
| 3:46414961 | 67.613 | 0 | 0.00 | 0 | -1 | 0.00 | 0 |
| 3:46414975 | 67.613 | -1 | 0.00 | 40 | -1 | 0.03 | 0 |
| 3:46415059 | 67.613 | 0 | 0.00 | 0 | -1 | 0.00 | 0 |
| 3:46415060 | 67.613 | 0 | 0.00 | 1 | -1 | 0.00 | 0 |
| 3:46415061 | 67.613 | 0 | 0.00 | 43 | -1 | 0.03 | 0 |
| 3:46415066 | 67.613 | 0 | 0.01 | 0 | -1 | 0.00 | 0 |
| 3:46415112 | 67.613 | 0 | 0.00 | 0 | -1 | 0.00 | 0 |
| 3:46415115 | 67.613 | 0 | 0.00 | 0 | -1 | 0.00 | 0 |
| 3:46415199 | 67.613 | 0 | 0.00 | 1 | -1 | 0.00 | 0 |
| 3:46415255 | 67.613 | 0 | 0.00 | 1 | -1 | 0.00 | 0 |
| 3:46415264 | 67.613 | 0 | 0.00 | 0 | -1 | 0.00 | 0 |
| 3:46415345 | 67.613 | 0 | 0.00 | 0 | -1 | 0.00 | 0 |
| 3:46415384 | 67.613 | 0 | 0.00 | 0 | -1 | 0.00 | 0 |
| 3:46415386 | 67.613 | 0 | 0.00 | 0 | -1 | 0.00 | 0 |
| 3:46415393 | 67.613 | 0 | 0.00 | 0 | -1 | 0.00 | 0 |
| 3:46415397 | 67.613 | 0 | 0.02 | 0 | -1 | 0.00 | 0 |
| 3:46415409 | 67.613 | 0 | 0.00 | 0 | -1 | 0.00 | 0 |
| 3:46415455 | 67.613 | 0 | 0.00 | 1 | -1 | 0.00 | 0 |
| 3:46415564 | 67.613 | 0 | 0.00 | 3 | -1 | 0.00 | 0 |
| 3:46415689 | 67.613 | 0 | 0.00 | 0 | -1 | 0.00 | 0 |
| 3:46415742 | 67.613 | 0 | 0.00 | 9 | -1 | 0.01 | 0 |
| 3:46415815 | 67.613 | -1 | 0.00 | 0 | -1 | 0.00 | 0 |
| 3:46416072 | 67.614 | 0 | 0.00 | 1 | -1 | 0.00 | 0 |
| 3:46416145 | 67.614 | 0 | 0.26 | 1 | -1 | 0.00 | 0 |
| 3:46416151 | 67.614 | 0 | 0.00 | 1 | -1 | 0.00 | 0 |
| 3:46416169 | 67.614 | 0 | 0.00 | 2 | -1 | 0.00 | 0 |
| 3:46416216 | 67.614 | 0 | 0.00 | 95 | -1 | 0.07 | 0 |
| 3:46416236 | 67.614 | 0 | 0.06 | 0 | -1 | 0.00 | 0 |
| 3:46416239 | 67.614 | 0 | 0.06 | 0 | -1 | 0.00 | 0 |
| 3:46416261 | 67.614 | 0 | 0.00 | 0 | -1 | 0.00 | 0 |
| 3:46416398 | 67.614 | -1 | 0.99 | 1330 | -1 | 1.00 | 0 |
| 3:46416470 | 67.614 | 0 | 0.06 | 575 | -1 | 0.43 | 0 |
| 3:46416508 | 67.614 | 0 | 0.00 | 2 | -1 | 0.00 | 0 |
| 3:46416543 | 67.614 | 0 | 0.00 | 6 | -1 | 0.00 | 0 |
| 3:46416598 | 67.614 | 0 | 0.01 | 0 | -1 | 0.00 | 0 |
| 3:46416618 | 67.614 | 0 | 0.05 | 0 | -1 | 0.00 | 0 |
| 3:46416686 | 67.614 | 0 | 0.00 | 20 | -1 | 0.02 | 0 |
| 3:46416851 | 67.614 | 0 | 0.05 | 0 | -1 | 0.00 | 0 |
| 3:46416855 | 67.614 | 0 | 0.00 | 1 | -1 | 0.00 | 0 |
| 3:46416879 | 67.614 | 0 | 0.00 | 2 | -1 | 0.00 | 0 |
| 3:46416883 | 67.614 | 0 | 0.00 | 0 | -1 | 0.00 | 0 |
| 3:46417058 | 67.614 | 0 | 0.00 | 2 | -1 | 0.00 | 0 |
| 3:46417069 | 67.614 | 0 | 0.00 | 24 | -1 | 0.02 | 0 |
| 3:46417166 | 67.614 | 0 | 0.00 | 1 | -1 | 0.00 | 0 |
| 3:46417224 | 67.614 | 0 | 0.00 | 3 | -1 | 0.00 | 0 |
| 3:46417231 | 67.614 | 0 | 0.03 | 1 | -1 | 0.00 | 0 |
| 3:46417312 | 67.614 | 0 | 0.30 | 657 | -1 | 0.49 | 0 |
| 3:46417326 | 67.614 | 0 | 0.00 | 5 | -1 | 0.00 | 0 |
| 3:46417340 | 67.614 | 0 | 0.00 | 3 | -1 | 0.00 | 0 |
| 3:46417482 | 67.614 | 0 | 0.00 | 2 | -1 | 0.00 | 0 |
| 3:46417525 | 67.614 | 0 | 0.00 | 2 | -1 | 0.00 | 0 |
| 3:46417868 | 67.614 | 0 | 0.00 | 21 | -1 | 0.02 | 0 |
