# Supplementary material for: Intragenus (Homo) variation in a chemokine receptor gene (CCR5)
Source: PLoS One. 2018 Oct 2;13(10):e0204989. doi: 10.1371/journal.pone.0204989 (PMC6168169; doi:10.1371/journal.pone.0204989)
Supplement: S2 Table — (DOCX) [file pone.0204989.s002.docx]

| **Supplementary Table 2: Variants in 1000 Genomes** | | | | | |  |
| --- | --- | --- | --- | --- | --- | --- |
| **RefSeq** | **Position (HG19)** | **Ref** | **Alt** | **Qual** | **Region** | **SNP-SUM** |
| rs543579626 | 46409683 | A | C | 100 | PU | 1 |
| rs565095660 | 46409690 | G | A | 100 | PU | 2 |
| rs192002630 | 46409728 | A | C | 100 | PU | 1 |
| rs547721330 | 46409731 | G | A | 100 | PU | 1 |
| rs559344601 | 46409741 | G | T | 100 | PU | 1 |
| rs529688443 | 46409742 | A | G | 100 | PU | 1 |
| rs547916512 | 46409794 | G | C | 100 | PU | 1 |
| rs140587963 | 46409935 | C | T | 100 | PU | 1 |
| rs536770486 | 46409973 | C | T | 100 | PU | 1 |
| rs41491345 | 46410020 | A | G | 100 | PU | 20 |
| rs7637813 | 46410036 | G | A | 100 | PU | 2377 |
| rs202002844 | 46410103 | T | C | 100 | PU | 2 |
| rs41490645 | 46410137 | A | C | 100 | PU | 208 |
| rs574835762 | 46410275 | G | A | 100 | PU | 1 |
| rs142620835 | 46410279 | G | A | 100 | PU | 4 |
| rs557102493 | 46410284 | C | T | 100 | PU | 1 |
| rs41499550 | 46410306 | T | C | 100 | PU | 326 |
| rs140523021 | 46410396 | C | G | 100 | PU | 7 |
| rs565029190 | 46410413 | G | C | 100 | PU | 1 |
| rs577113895 | 46410433 | G | A | 100 | PU | 1 |
| rs41386445 | 46410483 | C | T | 100 | PU | 3 |
| rs2856757 | 46410494 | A | C | 100 | PU | 1365 |
| rs41395049 | 46410499 | T | C | 100 | PU | 319 |
| rs41412948 | 46410507 | C | T | 100 | PU | 329 |
| rs563153875 | 46410517 | T | C | 100 | PU | 2 |
| rs76436340 | 46410531 | T | A | 100 | PU | 2 |
| rs551859885 | 46410728 | C | G | 100 | PU | 1 |
| rs149977098 | 46410746 | G | A | 100 | PU | 2 |
| rs146556912 | 46410768 | G | A | 100 | PU | 1 |
| rs546683708 | 46410864 | T | A | 100 | PU | 2 |
| rs41365248 | 46410921 | C | T | 100 | PU | 1 |
| rs535792329 | 46410925 | A | G | 100 | PU | 2 |
| rs191230884 | 46410932 | G | A | 100 | PU | 6 |
| rs2734225 | 46410936 | G | T | 100 | PU | 1342 |
| rs41458950 | 46410938 | G | A | 100 | PU | 14 |
| rs41469750 | 46410953 | G | A | 100 | PU | 11 |
| rs145146492 | 46410962 | T | C | 100 | PU | 14 |
| rs541210726 | 46411020 | G | A | 100 | PU | 2 |
| rs553230690 | 46411083 | C | T | 100 | PU | 2 |
| rs139018793 | 46411115 | T | C | 100 | PU | 1 |
| rs41475349 | 46411133 | G | A | 100 | PU | 319 |
| rs143788172 | 46411174 | T | C | 100 | PU | 1 |
| rs530424450 | 46411196 | G | T | 100 | PU | 1 |
| rs148007422 | 46411200 | G | A | 100 | PU | 3 |
| rs563823814 | 46411260 | T | C | 100 | PU | 1 |
| rs528203079 | 46411315 | C | T | 100 | PU | 1 |
| rs41384848 | 46411369 | C | T | 100 | PU | 8 |
| rs568574805 | 46411370 | G | A | 100 | PU | 4 |
| rs529547367 | 46411408 | G | C | 100 | PU | 1 |
| rs550991942 | 46411432 | C | T | 100 | PU | 1 |
| rs569580492 | 46411486 | T | A | 100 | PU | 1 |
| rs539832725 | 46411493 | T | A | 100 | PU | 2 |
| rs186785168 | 46411530 | A | T | 100 | PU | 1 |
| rs2227010 | 46411542 | G | A | 100 | PU | 2275 |
| rs534304693 | 46411544 | C | T | 100 | PU | 2 |
| rs565628795 | 46411556 | A | G | 100 | PU | 8 |
| rs2856758 | 46411661 | A | G | 100 | PU | 257 |
| rs542055812 | 46411679 | T | A | 100 | UTR5 | 1 |
| rs556969790 | 46411741 | G | C | 100 | Intron | 1 |
| rs145751198 | 46411755 | A | C | 100 | PD | 2 |
| rs545373945 | 46411803 | C | T | 100 | PD | 1 |
| rs563762909 | 46411804 | G | A | 100 | PD | 1 |
| rs138621551 | 46411817 | T | G | 100 | PD | 2 |
| rs2734648 | 46411840 | G | T | 100 | PD | 1630 |
| rs192425331 | 46411846 | G | A | 100 | PD | 1 |
| rs1799987 | 46411935 | A | G | 100 | PD | 1980 |
| rs551196542 | 46411957 | A | T | 100 | PD | 1 |
| rs569433974 | 46411963 | C | T | 100 | PD | 2 |
| rs533695265 | 46411973 | T | C | 100 | PD | 4 |
| rs551771466 | 46412004 | T | C | 100 | PD | 1 |
| rs41339446 | 46412005 | A | G | 100 | PD | 8 |
| rs534240555 | 46412082 | A | G | 100 | PD | 1 |
| rs549206526 | 46412087 | C | T | 100 | PD | 2 |
| rs28620009 | 46412138 | A | G | 100 | PD | 13 |
| rs142710698 | 46412149 | C | T | 100 | PD | 1 |
| rs557149889 | 46412184 | C | T | 100 | PD | 1 |
| rs575169866 | 46412188 | C | A | 100 | PD | 1 |
| rs147420214 | 46412244 | A | G | 100 | PD | 7 |
| rs1799988 | 46412259 | C | T | 100 | PD | 1980 |
| rs41469351 | 46412262 | C | T | 100 | PD | 307 |
| rs540267036 | 46412267 | C | T | 100 | PD | 1 |
| rs1800023 | 46412308 | A | G | 100 | PD | 1344 |
| rs573714896 | 46412427 | T | C | 100 | PD | 1 |
| rs544455720 | 46412434 | G | T | 100 | PD | 1 |
| rs563118331 | 46412454 | C | T | 100 | PD | 1 |
| rs533626678 | 46412473 | C | T | 100 | UTR5 | 2 |
| rs552107409 | 46412479 | G | T | 100 | UTR5 | 1 |
| rs560812992 | 46412496 | C | A | 100 | Intron | 1 |
| rs527678371 | 46412550 | C | T | 100 | Intron | 1 |
| rs149509262 | 46412554 | G | A | 100 | Intron | 3 |
| rs1800024 | 46412559 | C | T | 100 | Intron | 762 |
| rs187838508 | 46412585 | C | T | 100 | Intron | 2 |
| rs550471410 | 46412620 | A | G | 100 | Intron | 1 |
| rs568718768 | 46412648 | C | T | 100 | Intron | 2 |
| rs9282632 | 46412708 | A | C | 100 | Intron | 285 |
| rs144255721 | 46412753 | G | T | 100 | nc-transcript-variant | 3 |
| rs572840507 | 46412803 | G | A | 100 | nc-transcript-variant | 1 |
| rs533537621 | 46412871 | G | A | 100 | nc-transcript-variant | 4 |
| rs3181037 | 46412930 | A | G | 100 | Intron | 285 |
| rs193039140 | 46412978 | A | G | 100 | Intron | 3 |
| rs544310461 | 46413050 | G | T | 100 | Intron | 7 |
| rs563025816 | 46413061 | G | A | 100 | Intron | 1 |
| rs185207041 | 46413064 | G | T | 100 | Intron | 1 |
| rs547959533 | 46413068 | G | A | 100 | Intron | 9 |
| rs573333280 | 46413124 | C | G | 100 | Intron | 5 |
| rs528022257 | 46413134 | C | G | 100 | Intron | 1 |
| rs549083541 | 46413138 | G | A | 100 | Intron | 1 |
| rs561352865 | 46413150 | G | T | 100 | Intron | 1 |
| rs189663620 | 46413181 | C | T | 100 | Intron | 1 |
| rs550248476 | 46413201 | C | T | 100 | Intron | 1 |
| rs571452708 | 46413210 | T | C | 100 | Intron | 1 |
| rs532949499 | 46413235 | G | A | 100 | Intron | 1 |
| rs2856762 | 46413334 | C | T | 100 | Intron | 324 |
| rs2254089 | 46413418 | C | T | 100 | Intron | 1346 |
| rs41395249 | 46413419 | G | A | 100 | Intron | 50 |
| rs555028689 | 46413430 | G | C | 100 | Intron | 1 |
| rs192798693 | 46413439 | G | A | 100 | Intron | 1 |
| rs537491506 | 46413460 | G | T | 100 | Intron | 1 |
| rs183753875 | 46413623 | C | G | 100 | Intron | 2 |
| rs188423028 | 46413632 | G | A | 100 | Intron | 6 |
| rs143602732 | 46413655 | C | G | 100 | Intron | 4 |
| rs181867134 | 46413676 | G | T | 100 | Intron | 11 |
| rs2856764 | 46413743 | C | T | 100 | Intron | 1345 |
| rs186352754 | 46413781 | G | A | 100 | Intron | 1 |
| rs190462461 | 46413798 | G | A | 100 | Intron | 3 |
| rs531725192 | 46413802 | G | A | 100 | Intron | 1 |
| rs543450476 | 46413836 | A | G | 100 | Intron | 1 |
| rs140653397 | 46413889 | G | A | 100 | Intron | 3 |
| rs532495909 | 46413891 | G | T | 100 | Intron | 1 |
| rs150450395 | 46413932 | T | C | 100 | Intron | 13 |
| rs145371166 | 46413943 | C | T | 100 | Intron | 9 |
| rs2856765 | 46413950 | G | A | 100 | Intron | 1344 |
| rs549257838 | 46414023 | A | G | 100 | Intron | 1 |
| rs567947618 | 46414034 | A | G | 100 | Intron | 2 |
| rs41515644 | 46414035 | A | G | 100 | Intron | 1343 |
| rs556224798 | 46414109 | G | A | 100 | Intron | 7 |
| rs571339706 | 46414116 | T | C | 100 | Intron | 1 |
| rs538757349 | 46414169 | G | A | 100 | Intron | 5 |
| rs554369038 | 46414176 | G | A | 100 | Intron | 1 |
| rs181641346 | 46414199 | A | G | 100 | Intron | 1 |
| rs3176763 | 46414281 | G | T | 100 | Intron | 285 |
| rs41352147 | 46414282 | G | A | 100 | Intron | 17 |
| rs191374074 | 46414339 | A | G | 100 | Intron | 2 |
| rs182646932 | 46414349 | A | G | 100 | Intron | 8 |
| rs184985767 | 46414384 | G | C | 100 | Intron | 18 |
| rs200209014 | 46414436 | T | C | 100 | ORF | 1 |
| rs145061115 | 46414451 | T | A | 100 | ORF | 1 |
| rs189601230 | 46414470 | A | G | 100 | ORF | 2 |
| rs56340326 | 46414485 | G | A | 100 | ORF | 2 |
| rs113471490 | 46414498 | G | A | 100 | ORF | 1 |
| rs41425744 | 46414529 | G | A | 100 | ORF | 6 |
| rs531662647 | 46414536 | A | G | 100 | ORF | 1 |
| rs1799863 | 46414557 | T | A | 100 | ORF | 46 |
| rs142829420 | 46414580 | A | T | 100 | ORF | 1 |
| rs199722561 | 46414593 | T | C | 100 | ORF | 1 |
| rs547510206 | 46414598 | C | G | 100 | ORF | 1 |
| rs56198941 | 46414611 | C | T | 100 | ORF | 1 |
| rs1800941 | 46414618 | T | C | 100 | ORF | 9 |
| rs1800560 | 46414696 | T | A | 100 | ORF | 2 |
| rs183662584 | 46414709 | G | A | 100 | ORF | 6 |
| rs138174483 | 46414712 | C | T | 100 | ORF | 3 |
| rs558483026 | 46414720 | T | G | 100 | ORF | 1 |
| rs146209111 | 46414766 | G | A | 100 | ORF | 1 |
| rs541069027 | 46414781 | G | A | 100 | ORF | 4 |
| rs559783442 | 46414783 | C | T | 100 | ORF | 4 |
| rs574865763 | 46414852 | G | T | 100 | ORF | 3 |
| rs542780938 | 46414861 | T | C | 100 | ORF | 1 |
| rs55639502 | 46414870 | G | A | 100 | ORF | 2 |
| rs199824195 | 46414925 | T | C | 100 | ORF | 3 |
| rs201797884 | 46414961 | T | C | 100 | ORF | 1 |
| rs139737901 | 46415011 | G | A | 100 | ORF | 1 |
| rs201290940 | 46415059 | T | G | 100 | ORF | 1 |
| rs200419576 | 46415060 | C | T | 100 | ORF | 1 |
| rs1800452 | 46415061 | G | A | 100 | ORF | 60 |
| rs146972949 | 46415066 | C | T | 100 | ORF | 2 |
| rs548727619 | 46415074 | G | A | 100 | ORF | 1 |
| rs150592242 | 46415255 | A | G | 100 | ORF | 2 |
| rs537300448 | 46415293 | C | A | 100 | ORF | 1 |
| rs55916127 | 46415294 | G | A | 100 | ORF | 1 |
| rs188772198 | 46415345 | A | G | 100 | ORF | 2 |
| rs534677585 | 46415361 | G | T | 100 | ORF | 1 |
| rs199846907 | 46415384 | G | C | 100 | ORF | 1 |
| rs201780857 | 46415386 | T | C | 100 | ORF | 1 |
| rs1800944 | 46415397 | C | T | 100 | ORF | 65 |
| rs1800945 | 46415409 | A | T | 100 | ORF | 14 |
| rs543680286 | 46415414 | C | T | 100 | ORF | 1 |
| rs191297617 | 46415455 | C | T | 100 | UTR3 | 3 |
| rs532474349 | 46415471 | G | A | 100 | UTR3 | 1 |
| rs540992448 | 46415473 | T | A | 100 | UTR3 | 1 |
| rs536872608 | 46415527 | G | A | 100 | UTR3 | 3 |
| rs529752751 | 46415561 | T | A | 100 | UTR3 | 1 |
| rs146080174 | 46415564 | C | G | 100 | UTR3 | 7 |
| rs569458865 | 46415637 | C | T | 100 | UTR3 | 3 |
| rs552382275 | 46415734 | C | T | 100 | UTR3 | 1 |
| rs570782804 | 46415737 | C | T | 100 | UTR3 | 5 |
| rs41414147 | 46415742 | G | A,C | 100 | UTR3 | 61 |
| rs568368266 | 46415784 | A | T | 100 | UTR3 | 1 |
| rs535892345 | 46415813 | T | C | 100 | UTR3 | 6 |
| rs143589063 | 46415815 | C | T | 100 | UTR3 | 2 |
| rs575840249 | 46415870 | T | C | 100 | UTR3 | 9 |
| rs543618933 | 46415910 | G | T | 100 | UTR3 | 2 |
| rs369515918 | 46415951 | T | C | 100 | UTR3 | 2 |
| rs577174845 | 46416010 | G | T | 100 | UTR3 | 1 |
| rs540976580 | 46416050 | A | G | 100 | UTR3 | 1 |
| rs145725153 | 46416072 | T | A | 100 | UTR3 | 1 |
| rs373926603 | 46416104 | T | C | 100 | UTR3 | 1 |
| rs541845196 | 46416105 | G | T | 100 | UTR3 | 5 |
| rs41495153 | 46416145 | G | A | 100 | UTR3 | 286 |
| rs142957506 | 46416151 | G | A | 100 | UTR3 | 2 |
| rs151122728 | 46416169 | G | A | 100 | UTR3 | 3 |
| rs570721731 | 46416202 | C | T | 100 | UTR3 | 1 |
| rs528738443 | 46416203 | G | A | 100 | UTR3 | 1 |
| rs17765882 | 46416216 | C | T | 100 | UTR3 | 323 |
| rs41418945 | 46416236 | G | A | 100 | UTR3 | 77 |
| rs41466044 | 46416239 | G | A | 100 | UTR3 | 77 |
| rs190456231 | 46416261 | G | A | 100 | UTR3 | 1 |
| rs569344707 | 46416311 | A | G | 100 | UTR3 | 3 |
| rs539953254 | 46416312 | G | C | 100 | UTR3 | 1 |
| rs41345848 | 46416396 | G | A | 100 | UTR3 | 2 |
| rs141257116 | 46416398 | A | G | 100 | UTR3 | 2 |
| rs1800874 | 46416470 | G | T | 100 | UTR3 | 1342 |
| rs201423367 | 46416496 | C | G | 100 | UTR3 | 8 |
| rs186889269 | 46416508 | C | T | 100 | UTR3 | 2 |
| rs146926765 | 46416543 | G | C | 100 | UTR3 | 14 |
| rs562961861 | 46416562 | G | A | 100 | UTR3 | 1 |
| rs574954754 | 46416583 | G | A | 100 | UTR3 | 1 |
| rs137866928 | 46416598 | G | T | 100 | UTR3 | 4 |
| rs41535253 | 46416618 | T | C | 100 | UTR3 | 67 |
| rs528671807 | 46416656 | C | T | 100 | UTR3 | 1 |
| rs41526948 | 46416686 | A | G | 100 | UTR3 | 33 |
| rs562220468 | 46416752 | A | G | 100 | UTR3 | 2 |
| rs529611447 | 46416753 | C | T | 100 | UTR3 | 2 |
| rs550958125 | 46416774 | A | G | 100 | UTR3 | 1 |
| rs3188094 | 46416851 | A | C | 100 | UTR3 | 66 |
| rs190094030 | 46416855 | G | A | 100 | UTR3 | 3 |
| rs551901023 | 46416861 | C | T | 100 | UTR3 | 1 |
| rs185327310 | 46416883 | C | G | 100 | UTR3 | 1 |
| rs534497551 | 46416926 | G | A | 100 | UTR3 | 1 |
| rs553155294 | 46416951 | A | T | 100 | UTR3 | 1 |
| rs141737357 | 46417058 | G | T | 100 | UTR3 | 1 |
| rs41442546 | 46417069 | C | A | 100 | UTR3 | 71 |
| rs556971174 | 46417157 | G | T | 100 | UTR3 | 2 |
| rs574892806 | 46417163 | G | A | 100 | UTR3 | 2 |
| rs190136403 | 46417166 | G | A | 100 | UTR3 | 25 |
| rs376306118 | 46417172 | G | A | 100 | UTR3 | 3 |
| rs182052831 | 46417224 | C | T | 100 | UTR3 | 1 |
| rs41512547 | 46417231 | C | G | 100 | UTR3 | 46 |
| rs568854883 | 46417266 | G | T | 100 | UTR3 | 1 |
| rs529519962 | 46417290 | G | A | 100 | UTR3 | 1 |
| rs551297914 | 46417299 | T | G | 100 | UTR3 | 1 |
| rs746492 | 46417312 | G | T | 100 | UTR3 | 1994 |
| rs186138215 | 46417326 | T | G | 100 | UTR3 | 6 |
| rs192256035 | 46417340 | A | T | 100 | UTR3 | 3 |
| rs566824961 | 46417362 | G | A | 100 | UTR3 | 1 |
| rs534316499 | 46417477 | T | C | 100 | UTR3 | 1 |
| rs184040468 | 46417482 | G | A | 100 | UTR3 | 1 |
| rs370386700 | 46417498 | G | A | 100 | UTR3 | 2 |
| rs535314315 | 46417504 | A | C | 100 | UTR3 | 1 |
| rs570463527 | 46417509 | G | A | 100 | UTR3 | 3 |
| rs188577829 | 46417525 | T | G | 100 | UTR3 | 10 |
