# Supplementary material for: Intragenus (Homo) variation in a chemokine receptor gene (CCR5)
Source: PLoS One. 2018 Oct 2;13(10):e0204989. doi: 10.1371/journal.pone.0204989 (PMC6168169; doi:10.1371/journal.pone.0204989)
Supplement: S3 Table — (DOCX) [file pone.0204989.s003.docx]

| **Supplementary Table 3: Raw counts of variants per sample** | | | | | |  |  |  |
| --- | --- | --- | --- | --- | --- | --- | --- | --- |
|  | refseq | 1kg | alt | den3 | den2 | mez | ust | vin |
| ORF | 1059 | 46 | 0 | 0 | 11 | 2 | 0 | 0 |
| PU | 2009 | 57 | 3 | 5 | 5 | 10 | 4 | 3 |
| PD (contained in ORF) | 709 | 26 | 0 | 0 | 8 | 1 | 0 | 0 |
